# Thermoelectric Generator Based on Kesterite (Cu_2_ZnSnS_4_) Synthesized via Sol–Gel Method

**DOI:** 10.3390/ma19101971

**Published:** 2026-05-10

**Authors:** Afef Tarhouni, Marcelo Augusto Malagutti, Tanguy Bernard, Narges Ataollahi, Eleonora Isotta, Andrea Chiappini, Hassen Dahman, Lassaad El Mir, Paolo Scardi

**Affiliations:** 1 LaphyMNE Laboratory, Faculty of Sciences of Gabes, University of Gabes, Gabes 6072, Tunisia; afeftarhouni2022@gmail.com (A.T.); hassen.dahman@univgb.tn (H.D.); lassaad.elmir@fsg.rnu.tn (L.E.M.); 2 Department of Civil, Environmental and Mechanical Engineering, University of Trento, Via Mesiano 77, 38123 Trento, Italy; marcelo.malagutti@unitn.it (M.A.M.); tanguy.bernard@unitn.it (T.B.); narges.ataollahi@unitn.it (N.A.); eleonora.isotta@gmail.com (E.I.); 3 CSMFO Laboratory, Fondazione Bruno Kessler (FBK) Photonics Unit, Institute of Photonics and Nanotechnologies (IFN-CNR), Via Alla Cascata 56/C, 39123 Trento, Italy; andrea.chiappini@cnr.it

**Keywords:** CZTS, thermoelectric generator, sol–gel, ball milling, doctor blade, Seebeck coefficient, power factor

## Abstract

The need for sustainable and cost-effective thermoelectric materials has brought attention to earth-abundant and mineral compounds, like Cu_2_ZnSnS_4_ (CZTS). In this work, CZTS nanoparticles (NPs) were synthesized via the sol–gel method using environmentally friendly solvents based on water and ethanol mixtures. The resulting CZTS NPs were then processed into inks through ball milling to produce a thin-film thermoelectric generator (TEG). Structural and microstructural properties were investigated via X-ray diffraction and Raman spectroscopy, confirming the kesterite CZTS phase upon sintering. The chalcogenide exhibited *p*-type semiconductor behaviour, with a Seebeck coefficient reaching ~69 µV/K at 385 K. Van-der-Pauw measurements of conductivity confirmed a non-degenerate semiconducting behaviour, achieving ~1.77 S/cm at 323 K. A two-leg CZTS thin-film TEG reaching a maximum power output of 32(3) nW at a ΔT ~160 K was used, measured with a home-made setup. The volume-specific power of the generator reached $4\times 10^{-4}\;$μW cm^−3^ K^−2^. These results point to an effective use of sol–gel-based techniques to produce a functional thermoelectric generator, providing a costless and environmentally friendly approach to CZTS NPs.

## 1. Introduction

Kesterite Cu_2_ZnSnS_4_ (CZTS) is a quaternary semiconductor that was initially developed as a sustainable alternative to Cu(In,Ga)Se_2_ absorbers in thin-film photovoltaic devices [1]. In contrast to CIGS, CZTS is composed exclusively of earth-abundant and environmentally benign elements (Cu, Zn, Sn, and S) [2], exhibiting a high absorption coefficient (α > 10^4^ cm^−1^) and a near-optimal direct band gap of approximately 1.5 eV [3,4,5,6]. For this reason, recent advances in solution processing and interface engineering have led to CZTS-based solar cells with efficiencies reaching 15.1% [7]. Beyond photovoltaics, CZTS belongs to the broader family of copper-based chalcogenides investigated as efficient and potentially low-cost thermoelectric materials [8,9,10,11,12], offering a more sustainable alternative to established systems such as Bi_2_Te_3_, Sb_2_Te_3_, PbTe and GeTe, that depend on scarce and expensive elements [13]. This interest is further supported by the fact that several key attributes of CZTS—its diamond-like structure, tuneable electronic band structure, and intrinsically low lattice thermal conductivity—are also advantageous for thermoelectric energy conversion [13,14].

The thermoelectric performance of a material is quantified by the dimensionless figure of merit

(1) $$zT=\frac{S^{2}\sigma }{\kappa }\;T$$

where *S* is the Seebeck coefficient (μV K^−1^), *σ* the electrical conductivity (S cm^−1^), *κ* the total thermal conductivity (W m^−1^ K^−1^), and *T* (K) the absolute temperature [15,16].

Initial efforts to maximize zT focused on bulk CZTS synthesized by mechanochemical routes. Baláž et al. [17] demonstrated the semi-industrial synthesis of Cu-based chalcogenides using an eccentric vibratory mill, reporting a maximum zT of 0.04 at 673 K for bulk CZTS. Subsequent studies showed that thermoelectric performance can be optimized through control of cation disorder, particularly by promoting the partially disordered tetragonal kesterite phase (space group *I*-42m) [18,19], which modifies carrier scattering and transport properties. By this route, further disorder can be achieved by stabilizing the cubic sphalerite phase (*F*-43m) with complete cation disorder [19,20,21,22,23].

More recently, attention has shifted toward thin-film thermoelectric devices, where optimization of the power factor (*S*^2^*σ*) is particularly critical. For example, Kumar et al. [24] deposited CZTS thin films by chemical vapour deposition at different temperatures and reported a significant increase in power factor from 2 to 7 µW K^−2^ m^−1^ with increasing deposition temperature. This enhancement was attributed to changes in carrier concentration associated with secondary phase formation.

Thin-film devices based on CZTS and related kesterites have demonstrated promising performance per unit cost, highlighting their potential for scalable thermoelectric applications [13]. Various deposition techniques have been explored for CZTS thin films, including sputtering, hot injection, ball milling-based ink processing, and spin coating [14,25]. While sputtering yields high phase purity and excellent stoichiometric control, it involves complex and costly equipment. Alternative solution-based approaches offer advantages in scalability and cost-effectiveness, though they often face challenges related to phase control, contamination, and particle size distribution [14,25].

Among these approaches, the sol–gel method represents a particularly attractive route due to its simplicity, compositional flexibility, and ability to produce homogeneous thin films from low-cost precursors [26]. Importantly, sol–gel processing enables precise control over stoichiometry and defect chemistry, which are key parameters governing charge transport in CZTS [27]. Previous studies have shown that adjusting sulphur content improves both the Seebeck coefficient and electrical conductivity by enhancing phase purity and reducing detrimental sulphur-related defects. Similarly, tuning the Sn precursor concentration has been demonstrated to significantly modify carrier concentration and transport behaviour, highlighting the strong influence of cation stoichiometry on intrinsic defect formation and scattering mechanisms [28]. In addition to compositional control, optimization of film thickness and microstructure through spin-coating strategies has been reported to enhance grain connectivity and reduce interfacial scattering, thereby improving the power factor [29]. Collectively, these findings demonstrate that sol–gel processing provides a versatile platform for tailoring composition and microstructure to optimize the thermoelectric performance of CZTS thin films.

The aim of this work is to fabricate a TE device based on sustainable CZTS material. We adopt a simple and environmentally benign sol–gel strategy using distilled water and ethanol as eco-friendly solvents for the synthesis of CZTS nanoparticles. These nanoparticles are subsequently formulated into inks for thin-film fabrication and thermoelectric device assembly. The structural, morphological, and electrical properties of the resulting CZTS-based thermoelectric generators are systematically investigated, with emphasis on the relationship between processing conditions, microstructure, and thermoelectric performance.

## 2. Experimental Section

### 2.1. Materials

Copper (II) chloride dihydrate (CuCl_2_·2H_2_O; 99% pure), zinc chloride (ZnCl_2_·2H_2_O; 98% pure), tin (II) chloride dihydrate (SnCl_2_·2H_2_O; 98% pure), thiourea (CH_4_N_2_S; 99%), Oleylamine (70% pure), sulphur (99.5% pure), and ethanol (96%) were obtained from Sigma-Aldrich (St. Louis, MO, USA). Distilled water was used for the synthesis.

### 2.2. Synthesis of CZTS NPs and TEG Fabrication

The experimental procedure utilized chloride precursors of copper, zinc, and tin, alongside thiourea as the sulphur source. To prevent precipitation during the process and ensure the preparation of a stoichiometric Cu_2_ZnSnS_4_ solution, a 50% excess of sulphur was employed. Each precursor was dissolved separately in a water–ethanol (70–30%) mixture for 15 min. Subsequently, all four precursors were mixed under magnetic stirring at 50 °C for 2 h, resulting in a homogeneous yellow solution. The CZTS solution was filtered using a glass funnel to obtain CZTS NPs. The filtered material was then dried in an oven at 150 °C for 1 h. Following the drying process, the CZTS NPs were ground into a fine powder and subjected to sulfurization at 350 °C for 4 h under an inert atmosphere. The overall synthesis reaction followed the equation below:

(2) $$2CuCl_{2}+ZnCl_{2}+SnCl_{4}+4\left(CH_{4}N_{2}S\right)\to Cu_{2}ZnSnS_{4}+byproducts$$

The CZTS inks were prepared by combining 0.5 g of CZTS NPs with 2.5 mL of Oleylamine. The mixture was milled in a Fritsch P4 planetary ball mill (Fritsch, Laufen, Germany) for 15 min, with a main disk rotation speed of the main disk of 300 rpm (main-disk-to-planet ratio of −1.8). The solvent was separated from the NPs using a centrifugation process (MPW-380, MED-Instruments, Jacksonville, FL, USA) at 12,000 rpm for 10 min, after washing the mixture with an ethanol/toluene solution (12.5 mL:0.5 mL). Subsequently, an additional 0.5 mL of toluene was introduced, and the resulting solution was subjected to a sonication bath for 15 min to ensure thorough dispersion.

**Figure 1:**
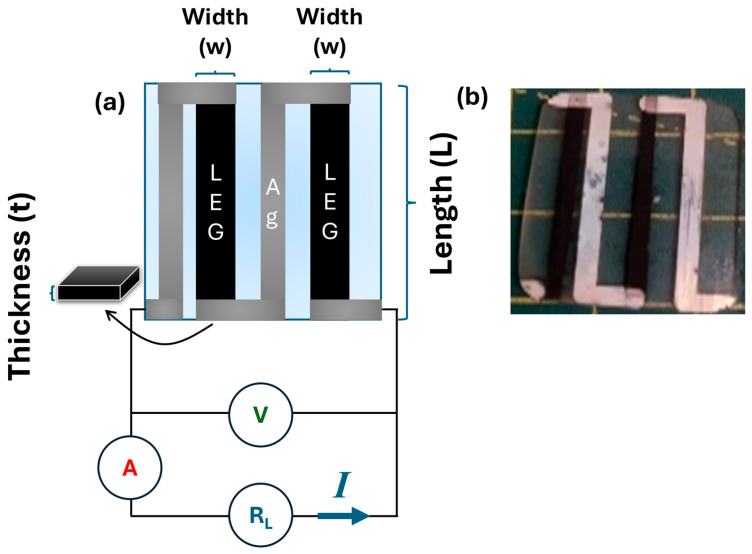
(**a**) Schematic representation of the thermoelectric generator (TEG) and the electrical circuit employed to acquire the current–voltage (I−V) characteristics. R_L_ denotes the external load resistance, A the ammeter used to measure the current, and V the voltmeter used to record the voltage. The dimensions of thickness, length, and width are indicated by the brackets and arrows. (**b**) Photograph of the CZTS-based TEG device used for the electrical measurements.

The CZTS thin-films were fabricated using the CZTS inks. First, soda-lime glass (SLG) substrates were cleaned. Then, a mask with specific dimensions was prepared using Kapton tape. Next, 0.1 mL of CZTS ink was deposited onto the substrates using a Finnpipette (Thermo Fisher Scientific, Waltham, MA, USA) to form the thin films by the doctor blade deposition method. Finally, the thin films underwent sulfurization at 400 °C for 1 h with a heating rate of 3 °C/min under a controlled atmosphere (N_2_), with ~85 mg of sulphur placed next to the sample. The thin films were patterned as shown in Figure 1a, connected by thermally evaporated Ag thin-film contacts (see [8] for details).

### 2.3. Characterization Techniques

The phase of the as-prepared CZTS NPs and thin films was investigated by X-ray diffraction (XRD Bruker D8 Discover, Bruker, Billerica, MA, USA) with CoKα radiation λ = 1.78901 Å in Bragg–Brentano geometry. Phase identification used the PDF4+ database [30] and Rietveld analysis was performed using TOPAS 7 software [31,32]. Whole-powder pattern modelling employed the macros developed by Scardi et al. [33,34,35] to estimate crystallite size and distribution. The fundamental parameters approach was employed to deconvolve the instrumental resolution function [36]. Baseline fitting employed Chebyshev functions.

The Raman measurements were performed using a LabRAM Aramis (Horiba Jobin-Yvon, Palaiseau, France), which has an optical microscope and 100 × objective, with a 532 nm source wavelength. The conductivity was estimated using Van der Pauw measurements performed with a Linseis HCS-1 instrument (Linseis, Selb, Germany).

For electrical characterization, a specifically shaped CZTS thin film with square dimensions of 12.5 mm × 12.5 mm with a thickness of 0.22 mm was fabricated, following the same conditions for the TEG deposition. Ohmic contact verification was performed before the measurement, varying the current from 0.1 to 1 mA for all 4 spring-load contacts, yielding a linear relationship between current and measured voltage. The magnetic field range was from −0.7 to 0.7 T, with measurements performed in a controlled N2 atmosphere at an applied current of 1 mA. The thickness of the samples was measured by a Dektak3 Stylus Profilometer (Veeco Instruments, Plainview, NY, USA).

The TEG’s power output was measured using a home setup to estimate the current–voltage–power curves (I−V−P). It consists of two thermocouples, one manually attached to two micro-heaters in the hot side, and another in the Peltier cooler to measure the cold side temperature. Upon generation of a temperature gradient by the micro-heaters, a load resistance connected in series with the TEG was varied from 0 to 2 MΩ using a newly developed automated system [8]. The voltage (V) and current (I) were measured with a Keithley 2601A source meter (Keithley Instruments Inc., Cleveland, OH, USA), connected simultaneously in parallel and in series, as represented in the circuit of Figure 1a. Calibration of the resistance applied was estimated by Ohm’s law (R=V/I). The power output was evaluated according to the standard relation: $P_{\mathrm{OUT}}=V\times I=R\times I^{2}$. To ensure a stable acquisition of current and voltage values, the measurements were repeated three times. The uncertainty was estimated from the instrument precision specified by the manufacturer.

## 3. Results and Discussion

### 3.1. Structural and Microstructural Characterization

**Figure 2:**
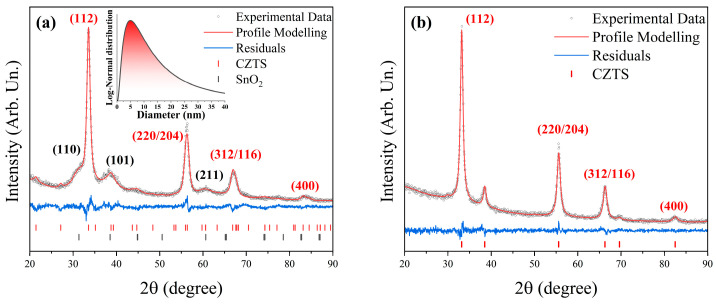
(**a**) XRD pattern and profile modelling of the CZTS NPs and (**b**) XRD pattern and profile modelling of the CZTS thin film. The black open dots represent the measured data, the red line the profile modelling, and the residuals are represented in blue. The black markers correspond to the Miller indices of the CZTS phase and the red ones to the SnO_2_ phase.

The XRD pattern of the as-prepared CZTS nanoparticles in Figure 2a confirms that the dominant crystalline phase corresponds to tetragonal kesterite CZTS (space group 121, *I*-42m, PDF #00-026-0575), representing approximately 73(3) wt% of the sample. A secondary phase identified as cassiterite SnO_2_ (space group 136, *P*_42_/mnm, PDF #01-070-4176) is also detected. The occupation of Cu atoms at the x = 0, y = 1/2, and z = 1/4 positions and Sn atoms was refined, yielding values of 0.15 and 0.6, respectively, indicating that the CZTS structure is Cu- and Sn-deficient. The refined lattice parameters are a= 5.390(1) Å and c= 10.648(4)Å and the crystallite size is 9(2) nm on average with a standard deviation of 4(1) for a log-normal distribution as shown in the inset of Figure 2a. The value of the microstrain is $e_{0}=0.15(2)$ %.

After ink preparation, washing, and sulfurization, the XRD pattern of the CZTS legs, shown in Figure 2b, no longer exhibits detectable SnO_2_ reflections. The reduction of the oxide phase may originate from multiple mechanisms. First, SnO_2_ nanoparticles are known to interact with long-chain amine ligands such as Oleylamine (OLA), which can coordinate to metal-oxide surfaces and promote dispersion in organic media; ligand-assisted stabilization and phase transfer of SnO_2_ nanocrystals in nonpolar solvents has been reported in the literature [37]. Such interactions may facilitate preferential removal of oxide-rich fractions during solvent washing and centrifugation. Second, SnO_2_ can undergo sulfurization at moderate temperatures (≈350–450 °C), leading to the formation of SnS_2_ under sulphur-rich conditions [38]. In multinary chalcogenide systems, transient SnS_2_ has been reported to react further with Cu–S and Zn–S intermediates during annealing, contributing to the formation of kesterite CZTS [39]. Therefore, the disappearance of SnO_2_ reflections after sulfurization may result from a combination of partial physical removal during ink purification and chemical transformation during annealing. No additional crystalline secondary phases are observed within the detection limit of XRD, indicating that any intermediate sulphide species, if formed, are either consumed during the reaction or present below the instrumental sensitivity threshold.

**Figure 3:**
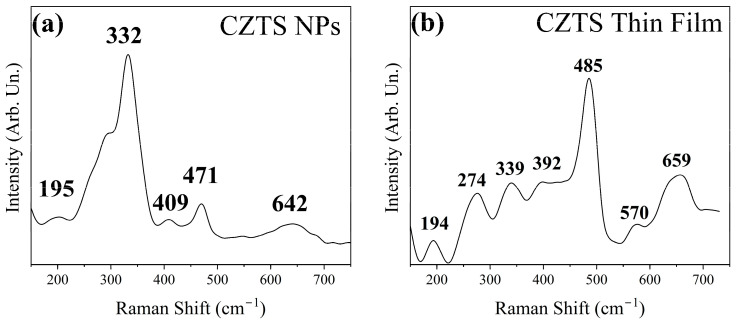
Raman spectrum of CZTS NPs (**a**) and CZTS thin film (**b**).

Raman spectroscopy provides complementary information on short-range order and minor phases. The nanoparticle (NP) spectrum (Figure 3a) is dominated by the characteristic A_1_ mode of kesterite CZTS, observed at ~332 cm^−1^, slightly downshifted from the typical 337–338 cm^−1^ position [21,40,41]. This shift is consistent with nanoscale effects and cation disorder, in agreement with the refined crystallite size and microstrain [11]. A weak feature near ~195 cm^−1^ further supports the presence of the kesterite phase. A small band at ~471 cm^−1^ suggests a minor Cu_2−x_S contribution, which may remain undetected by XRD due to its low concentration or limited crystallinity [24]. A weak band at ~642 cm^−1^ is consistent with Sn–O-related vibrations [42], corroborating the SnO_2_ fraction observed in the NP XRD pattern.

The Raman spectrum of the CZTS leg, in Figure 3b, exhibits kesterite-related modes at ~194, ~339, and ~392 cm^−1^, indicating improved crystallinity and structural ordering after annealing [41]. Additional bands at ~570 and ~659 cm^−1^ fall within the second-order (multiphonon) scattering region commonly reported for CZTS [43]. A pronounced band at ~485 cm^−1^ appears after processing. Since this feature does not correspond to the most commonly reported Raman signatures of ZnS, CTS, or SnS_x_ phases, it is tentatively attributed to disorder-activated or defect-related lattice vibrations, or possibly to local structural modifications induced during sulfurization or laser excitation. Further power-dependent Raman measurements would be required to conclusively determine its origin.

Importantly, the Raman spectrum of the thin film shows an intense broad band centred near ~1400 cm^−1^ (see Figure S1 of the Supporting Information File). This feature is characteristic of organic ligands, such as OLA, associated with CH_2_ bending and C–C vibrational modes [44]. The high Raman activity of organic species explains their strong spectral contribution, even when present in relatively small amounts. In contrast, XRD is largely insensitive to such organic residues due to their low electron density and poor crystallinity. Therefore, while XRD confirms the predominance of crystalline kesterite CZTS after processing, Raman spectroscopy reveals that residual organic ligands remain in the film and contribute significantly to the vibrational spectrum.

Overall, the combined XRD and Raman analyses indicate that the sulfurized films are predominantly kesterite CZTS, with oxide impurities substantially reduced after ink preparation and annealing. Minor sulphide-related species and residual organic ligands may persist but remain below the detection limit of XRD.

### 3.2. Electrical Conductivity

**Figure 4:**
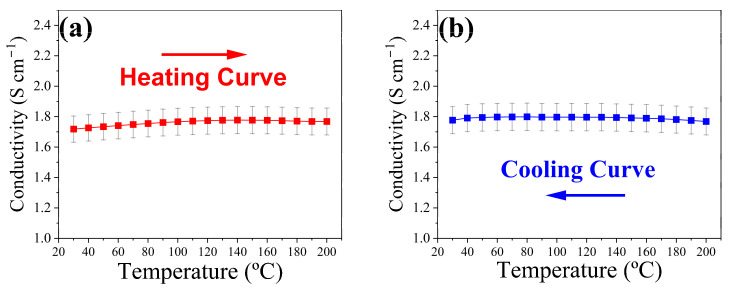
Electrical conductivity of the CZTS thin film measured in the Van der Pauw configuration during (**a**) heating and (**b**) cooling cycles.

The electrical conductivity (σ) of the CZTS thin film is shown in Figure 4. The overall variation in conductivity between room temperature and 200 °C is limited to approximately 3%, which is within the uncertainty range of the experimental measurement (5%). Above approximately 150 °C in the heating curves, a slight decrease in electrical conductivity is observed, which may be associated with disorder–order transitions in kesterite-type materials, as previously reported by Isotta et al. [45]. Upon cooling, the kinetics of this phase transformation are not observed, giving a constant value of conductivity throughout the entire temperature range. The results were compared with those reported for other chemical routes. The hot-injection ink-based TEGs developed by Syafiq et al. [14] reported an electrical conductivity of 0.28 S cm^−1^; the present CZTS thin film shows an approximately sixfold-higher value. However, Sharma and Neeleshwar observed higher electrical conductivity for CZTS-based TE materials reaching up to 2.1 S cm^−1^ [36]. Given the strong dependence of σ on the concentration of defects and on microstructural features, variations arising from different synthesis routes are to be expected, even in stoichiometric CZTS.

### 3.3. Performance Characteristics of the CZTS-Based TEG

Since the film displayed a measurable conductivity, it was employed as a thermoelectric generator, as shown in Figure 1. The thermoelectric performance of the device was evaluated using a custom-built measurement setup described elsewhere [8], in which both current and voltage were recorded simultaneously under controlled temperature gradients. Heating was applied to the hot side ($T_{hot}$) using resistive heaters positioned inside an insulated chamber, while the cold side was stabilized by a Peltier module ($T_{cold}$) to ensure a constant reference temperature. Before each measurement, sufficient time was allowed to establish a stable thermal gradient across the device. $T_{hot}\;$was varied from 323 K up to 473 K while maintaining the $T_{cold}\;$ 293 K.

**Figure 5:**
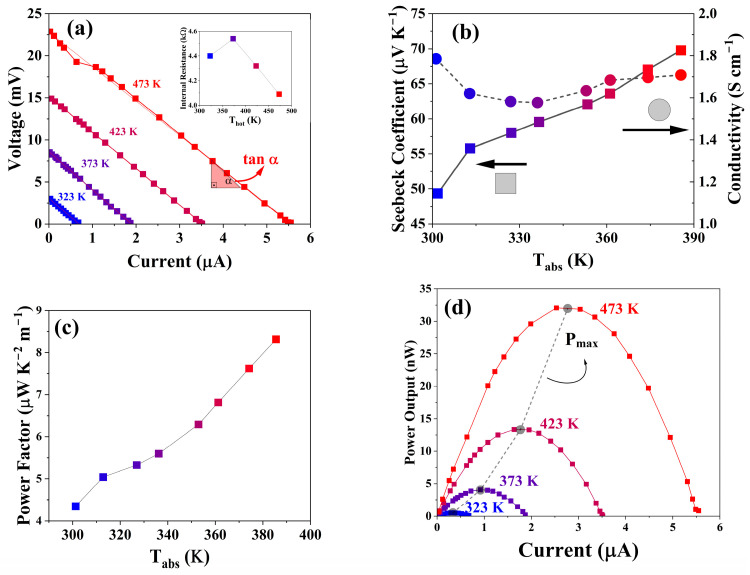
(**a**) I-V plots, (**b**) Seebeck (squares) and conductivity (spheres), (**c**) power factor, (**d**) power output obtained at different temperatures for the TEG. The maximum power output, $P_{max}$, is indicated by the dashed lines.

Figure 5a shows the current–voltage (I–V) characteristics obtained by systematically varying the external load resistance between 0 and 2 MΩ in discrete steps. The resulting I–V curves exhibit a linear relationship, confirming the Ohmic behaviour of the device and the absence of significant contact resistance. The internal resistance ($R_{int}$) of the TEG was extracted from the slope of the I–V curves ($R_{int}=\;-\Delta V/\Delta I$), yielding values on the order of 4 kΩ (see inset of Figure 5a). This internal resistance includes both intrinsic material resistance and contact contributions and therefore represents the effective resistance of the thermoelectric leg. Two limiting conditions define the electrical response: the open-circuit voltage (VOC), measured when the external resistance approaches infinity and the current is zero, and the short-circuit current ($I_{sc}$), measured when the external resistance is zero and the voltage vanishes.

The electrical conductivity (σ) of the CZTS thin film was recalculated from the internal resistance using the geometrical dimensions of the device according to $\sigma =L/(R_{int}\;A)$, where L is the leg length and A the cross-sectional area determined from the film width and thickness (values are reported in Table 1). The temperature dependence of the conductivity is shown in Figure 5b, exhibiting values similar to those of the Van-der-Pauw method in Figure 4. The conductivity can be considered stable through the entire range of temperature, given the intrinsic error of the setup instrument. This reveals that the contact resistance of the TEGs is minimal and does not deteriorate the electronic properties during the measurement.

The effective Seebeck coefficient was determined from the open-circuit voltage using S = (1/N)(VOC/ΔT), where N is the number of thermoelectric legs—2 in this case, and $\Delta T\;=\;T_{hot}\;-\;T_{cold}\;$is the applied temperature difference. The Seebeck coefficient increases from approximately 50 μV K^−1^ at 323 K to nearly 70 μV K^−1^ at 385 K, confirming the *p*-type semiconducting nature of CZTS through its positive sign (see Figure 5b). Although these values are lower than those reported for mechanically alloyed or cold-pressed CZTS materials [14,18], they remain consistent with thin-film kesterite systems.

The power factor ($PF=S^{2}\sigma$) was calculated to assess the ability of the CZTS film to generate useful electrical power and is given in Figure 5c. A gradual increase in PF with temperature is observed, reaching a maximum of approximately 8 nW cm^−1^ K^−2^ at the highest measurement temperature. While this value is lower than those reported for optimized bulk kesterite and CZTSe materials, it reflects the effect of the organic ligand, OLA, which is attributed to decreasing the conducting channels throughout the material. The relatively modest PF indicates that further defect engineering or carrier concentration optimization would be required to enhance performance.

The electrical power output was calculated from P=V I for each applied load resistance and is shown in Figure 5d. The power–current (I−P) curves exhibit the expected parabolic behaviour, with maximum power achieved when the external load resistance equals the internal resistance of the device. This condition is consistent with classical impedance matching in thermoelectric generators. The maximum power can also be estimated from $P_{max}\;=\;VOC\;I_{sc}/4$, reflecting the quadratic dependence of power on current. For the present CZTS TEG, a maximum output power of approximately 32(3) nW was obtained at the highest applied temperature gradient. Notably, the maximum power increases approximately with the square of the temperature difference ($P_{max}\;\propto \;\Delta T^{2}$), as expected from the proportionality of both VOC and $I_{sc}$ to ΔT. This quadratic scaling confirms stable thermoelectric behaviour without anomalous transport effects or contact limitations.

## 4. Discussion

To enable a meaningful comparison with literature reports on thermoelectric generators (TEGs), the maximum power output must be normalized with respect to both the applied temperature gradient and the characteristic length scale of the device, as discussed in [9,13]. Since the maximum output power scales approximately with ΔT^2^, performance comparisons are most appropriately carried out using $\Delta T^{2}$-normalized metrics. Two complementary normalization strategies are commonly employed: (i) normalization by the total device volume, yielding the volumetric temperature power density ($P_{volume}$, in W cm^−3^ K^−2^), and (ii) normalization by the active surface area, yielding the areal temperature power density ($P_{area}$, in W cm^−2^ K^−2^).

When applying this normalization to CZTS- and CZTSe-based thin-film TEGs reported in the literature, clear trends emerge in both volumetric and areal performance. For the ink-processed devices reported by Syafiq et al. [14] at ΔT = 160 K, the $\Delta T^{2}$-normalized volumetric power densities ($P_{volume}$) are 8.4 × 10^−3^ μW cm^−3^ K^−2^ for CZTS via hot injection, called HI-CZTS, (2.0 µm thick), 1.53 × 10^−2^ μW cm^−3^ K^−2^ for CZTSe via ball-milling, called BM-CZTSe (4.8 µm thick), 2.19 × 10^−3^ μW cm^−3^ K^−2^ for BM-CZTSSe (4.1 µm thick), and 4.53 × 10^−3^ μW cm^−3^ K^−2^ for BM-CTS (5.1 µm thick), confirming CZTSe as the volumetric champion within that study. In terms of areal-normalized performance ($P_{area}$), the same devices yield 1.68 × 10^−3^, 7.34 × 10^−3^, 8.98 × 10^−4^, and 2.30 × 10^−3^ nW cm^−2^ K^−2^ for HI-CZTS, BM-CZTSe, BM-CZTSSe, and BM-CTS, respectively [14]. A higher level of integration and improved contact engineering was reported by Isotta et al. [13], whose CZTS and CZTSe planar thin-film TEGs (ΔT = 150 K) exhibit $P_{volume}$ values of 2.72 × 10^−2^ and 4.13 × 10^−2^ μW cm^−3^ K^−2^, and $P_{area}$ values of 6.80 × 10^−3^ and 1.24 × 10^−2^ nW cm^−2^ K^−2^, respectively, representing a substantial improvement over early ink-based architectures. Overall, CZTSe devices consistently outperform CZTS counterparts in both volumetric and areal metrics, reflecting their higher electrical conductivity and optimized microstructure. Nevertheless, the differences between $P_{volume}$ and $P_{area}$ rankings highlight the strong influence of film thickness and device geometry: while volumetric normalization favours thinner, better-integrated films, areal normalization emphasizes lateral device design and scalability. These comparisons demonstrate that, although absolute performance remains below bulk thermoelectric benchmarks, thin-film CZTS and CZTSe TEGs fabricated through solution-based or low-cost routes already achieve competitive ΔT^2^-normalized power densities within the emerging class of sustainable chalcogenide generators.

In contrast, the present CZTS TEG, fabricated via a low-temperature sol–gel route followed by simple doctor-blade deposition and sulfurization, achieves a maximum power output of approximately 35 nW at ΔT~160 K. Normalizing by volume and temperature gradient squared, $P_{volume}=4\times 10^{-4}\;$μW cm^−3^ K^−2^, while by active surface area it achieves $P_{area}=3.5\times 10^{-4}\;$nW cm^−2^ K^−2^. Overall, while CZTSe and optimized CTS systems may deliver superior volumetric performance [14], CZTS thin films processed via cost-effective chemical routes demonstrate that functional nanowatt-level thermoelectric generation can be achieved without vacuum-based deposition or complex device architectures. From a materials sustainability and manufacturing perspective, the simplicity of the sol–gel approach represents an advantage, even if further optimization of carrier concentration and defect engineering is required to approach the highest reported device efficiencies.

## 5. Conclusions

In this work, CZTS thin films were successfully fabricated through a fully solution-based and environmentally benign route combining sol–gel synthesis of nanoparticles, ink formulation via ball milling, and doctor-blade deposition followed by sulfurization. The following results were achieved:

- Structural analysis confirmed the formation of predominantly tetragonal kesterite CZTS, while XRD and Raman spectroscopy evidenced the substantial reduction of secondary SnO_2_ phases after ink processing and annealing.
- Raman analysis further indicated improved structural ordering after sulfurization, although minor ligand residues and defect-related vibrational features remain detectable.
- Electrical transport measurements revealed stable *p*-type semiconducting behaviour, with a Seebeck coefficient reaching ~69 μV K^−1^ at 385 K and electrical conductivity up to 1.77 S cm^−1^.
- The resulting two-leg thin-film thermoelectric generator delivered a maximum output power of 32(3) nW under the applied temperature gradient, demonstrating reliable Ohmic behaviour and predictable $\Delta T^{2}$ scaling.
- The generator achieves competitive $\Delta T^{2}$-normalized volumetric and areal power densities compared with previously reported CZTS-based thin-film TEGs fabricated through more complex or vacuum-assisted approaches, when normalized to device geometry and temperature gradient.

Although the absolute power factor remains modest relative to optimized bulk or CZTSe-based systems, the present results demonstrate that functional thermoelectric generation can be achieved using low-temperature, scalable, and low-cost processing routes based entirely on earth-abundant elements. The simplicity of the sol–gel/ink-based strategy represents a significant advantage for sustainable thermoelectric device manufacturing. Further improvements are expected through optimization of carrier concentration, defect engineering, ligand removal, and microstructural densification. Overall, this study confirms that solution-processed CZTS thin films constitute a viable platform for environmentally friendly thermoelectric generators and provides a practical pathway toward scalable and sustainable chalcogenide-based energy harvesting devices.

## Figures and Tables

**Table 1:** TEG dimensions. t corresponds to the thickness, w to the width, L to the length, and A to the area of the device.

	t [µm]	w [cm]	L [cm]	A [cm^2^]
Leg A	94	0.143	2.5	1.35 × 10^−3^
Leg B	4.7	0.138	2.5	6.45 × 10^−5^

## Data Availability

The original contributions presented in this study are included in the article/Supplementary Material. Further inquiries can be directed to the corresponding author.

## Supplementary Materials

The following supporting information can be downloaded at https://www.mdpi.com/article/10.3390/ma19101971/s1. Figure S1: Raman spectra for the kesterite thin film (red line) and glass substrate (SiO_2_, given by the black line).

## References

[1] Rau U., Schock H.W. (1999). Electronic properties of Cu(In,Ga)Se_2_ heterojunction solar cells-recent achievements, current understanding, and future challenges. Appl. Phys. A.

[2] Ishii T., Otani K., Takashima T., Xue Y. (2013). Solar spectral influence on the performance of photovoltaic (PV) modules under fine weather and cloudy weather conditions. Prog. Photovolt. Res. Appl..

[3] Ziti A., Hartiti B., Labrim H., Fadili S., Batan A., Tahri M., Ridah A., Mounkachi O., Benyoussef A., Thevenin P. (2019). Characteristics of kesterite CZTS thin films deposited by dip-coating technique for solar cells applications. J. Mater. Sci. Mater. Electron..

[4] Ataollahi N., Bazerla F., Malerba C., Chiappini A., Ferrari M., Di Maggio R., Scardi P. (2019). Synthesis and Post-Annealing of Cu_2_ZnSnS_4_ Absorber Layers Based on Oleylamine/1-dodecanethiol. Materials.

[5] Ahmoum H., Sukor Su’ait M., Ataollahi N., Ubaidah Syafiq Mustaffa M., Boughrara M., Chelvanathan P., Sopian K., Li G., Kerouad M., Scardi P. (2021). Suppressing the secondary phases via N_2_ preheating of Cu_2_ZnSnS_4_ thin films with the addition of oleylamine and/or 1-Dodecanethiol solvents. Inorg. Chem. Commun..

[6] Su’ait M.S., Sahudin M.A., Ludin N.A., Ahmad A., Rahman M.Y.A., Ahmoum H., Ataollahi N., Scardi P. (2023). Potential of transition metal sulfides, Cu_2_ZnSnS_4_ as inorganic absorbing layers in dye-sensitized solar cells. J. Clean. Prod..

[7] Gong Y., Jimenez-Arguijo A., Caño I., Scaffidi R., Malerba C., Valentini M., Payno D., Navarro-Güell A., Segura-Blanch O., Flandre D. (2025). Attaining 15.1% Efficiency in Cu_2_ZnSnS_4_ Solar Cells Under Indoor Conditions Through Sodium and Lithium Codoping. Sol. RRL.

[8] Bernard T., Malagutti M.A., Lohani K., D’Incau M., Ataollahi N., Scardi P. (2024). Environmentally friendly p-type CTS-based thin-film thermoelectric generator. J. Mater. Sci..

[9] Malagutti M.A., Lohani K., Caño Prades I., Navarro-Güell A., Bernard T., Chiappini A., Saucedo E., Ataollahi N., Scardi P. (2025). CuFeS_2_/Cu_2_S/FeS_2_ Composite to Increase the Performance of Thin-Film Thermoelectric Generators Based on Sustainable Materials. ACS Appl. Electron. Mater..

[10] Malagutti M.A., Lohani K., D’Incau M., Nautiyal H., Ataollahi N., Scardi P. (2023). Optimizing CuFeS_2_ Chalcopyrite Thin Film Synthesis: A Comprehensive Three-Step Approach Using Ball-Milling, Thermal Evaporation, and Sulfurization Applied for Thermoelectric Generation. Appl. Sci..

[11] Nautiyal H., Lohani K., Mukherjee B., Isotta E., Malagutti M.A., Ataollahi N., Pallecchi I., Putti M., Misture S.T., Rebuffi L. (2023). Mechanochemical Synthesis of Sustainable Ternary and Quaternary Nanostructured Cu_2_SnS_3_, Cu_2_ZnSnS_4_, and Cu_2_ZnSnSe_4_ Chalcogenides for Thermoelectric Applications. Nanomaterials.

[12] Bernard T., Malagutti M.A., D’Incau M., Ataollahi N., Scardi P. (2025). Sustainable sulphides for π-type planar thermoelectric generators. MRS Energy Sustain..

[13] Isotta E., Andrade-Arvizu J., Syafiq U., Jiménez-Arguijo A., Navarro-Güell A., Guc M., Saucedo E., Scardi P. (2022). Towards Low Cost and Sustainable Thin Film Thermoelectric Devices Based on Quaternary Chalcogenides. Adv. Funct. Mater..

[14] Syafiq U., Isotta E., Ataollahi N., Lohani K., Luong S., Trifiletti V., Fenwick O., Scardi P. (2022). Facile and Low-Cost Fabrication of Cu/Zn/Sn-Based Ternary and Quaternary Chalcogenides Thermoelectric Generators. ACS Appl. Energy Mater..

[15] Goldsmid H.J. (2016). Introduction to Thermoelectricity.

[16] Lee H. (2016). Thermoelectrics: Design and Materials.

[17] Baláž P., Hegedüs M., Achimovičová M., Baláž M., Tešinský M., Dutková E., Kaňuchová M., Briančin J. (2018). Semi-industrial Green Mechanochemical Syntheses of Solar Cell Absorbers Based on Quaternary Sulfides. ACS Sustain. Chem. Eng..

[18] Isotta E., Syafiq U., Ataollahi N., Chiappini A., Malerba C., Luong S., Trifiletti V., Fenwick O., Pugno N., Scardi P. (2021). Thermoelectric properties of CZTS thin films: Effect of Cu-Zn disorder. Phys. Chem. Chem. Phys..

[19] Isotta E., Fanciulli C., Pugno N.M., Scardi P. (2019). Effect of the Order-Disorder Transition on the Seebeck Coefficient of Nanostructured Thermoelectric Cu_2_ZnSnS_4_. Nanomaterials.

[20] Bette S., Isotta E., Mukherjee B., Schulz A., Dallos Z., Kolb U., Dinnebier R.E., Scardi P. (2024). Microstructural Insights into the Transformation of Cubic, Low-Temperature, Disordered Cu_2_ZnSnS_4_ into the Tetragonal Form. J. Phys. Chem. C.

[21] Malagutti M.A., Isotta E., Bette S., Nautiyal H., Mukherjee B., Chiappini A., Smet J., Maduro Campos C.E., Dinnebier R., Ataollahi N. (2025). Investigating the Cubic-to-Tetragonal Phase Transition of Cu_2+y_Zn_1–y_SnS_x_Se_4–x_ Solid Solutions. Cryst. Growth Des..

[22] Isotta E., Mukherjee B., Bette S., Dinnebier R., Scardi P. (2022). Static and dynamic components of Debye–Waller coefficients in the novel cubic polymorph of low-temperature disordered Cu_2_ZnSnS_4_. IUCrJ.

[23] Mukherjee B., Isotta E., Fanciulli C., Ataollahi N., Scardi P. (2021). Topological Anderson Insulator in Cation-Disordered Cu_2_ZnSnS_4_. Nanomaterials.

[24] Kumar S., Ansari M.Z., Khare N. (2017). Enhanced thermoelectric power factor of Cu_2_ZnSnS_4_ in the presence of Cu_2−x_S and SnS_2_ secondary phase. AIP Conf. Proc..

[25] Chen D., Zhao Y., Chen Y., Wang B., Wang Y., Zhou J., Liang Z. (2015). Hot-Injection Synthesis of Cu-Doped Cu_2_ZnSnSe_4_ Nanocrystals to Reach Thermoelectric zT of 0.70 at 450 °C. ACS Appl. Mater. Interfaces.

[26] Zhou Z., Wang Y., Xu D., Zhang Y. (2010). Fabrication of Cu_2_ZnSnS_4_ screen printed layers for solar cells. Sol. Energy Mater. Sol. Cells.

[27] Ashfaq A., Jacob J., Mahmood K., Mehboob K., Ikram S., Ali A., Amin N., Hussain S., Rehman U. (2021). Effect of sulfur amount during post-growth sulfurization process on the structural, morphological and thermoelectric properties of sol-gel grown quaternary chalcogenide Cu_2_ZnSnS_4_ thin films. Phys. B Condens. Matter.

[28] Ali A., Jacob J., Ashfaq A., Mahmood K., Ahmad S., Rehman U., Ahmad W., Amin N., Ikram S., Hussain S. (2019). Effect of tin concentration on the structural, optical and thermoelectric properties of CZTS thin films gown by chemical solution method. Ceram. Int..

[29] Ahmoum H., Li G., Su’ait M.S., Boughrara M., Chelvanathan P., Khaaissa Y., Kerouad M., Wang Q. (2021). The impact of precursor thickness and surface roughness on the power factor of Cu_2_ZnSnS_4_ (CZTS) at near room temperature: Spin-coating deposition. Superlattices Microstruct..

[30] Gates-Rector S., Blanton T. (2019). The Powder Diffraction File: A quality materials characterization database. Powder Diffr..

[31] Coelho A.A. (2018). TOPAS and TOPAS-Academic: An optimization program integrating computer algebra and crystallographic objects written in C++. J. Appl. Crystallogr..

[32] (2009). TOPAS.

[33] Scardi P., Azanza Ricardo C.L., Perez-Demydenko C., Coelho A.A. (2018). Whole powder pattern modelling macros for TOPAS. J. Appl. Crystallogr..

[34] Scardi P. (2020). Diffraction Line Profiles in the Rietveld Method. Cryst. Growth Des..

[35] Scardi P., D’Incau M., Malagutti M.A., Terban M.W., Hinrichsen B., Fitch A.N. (2025). A reference material for X-ray diffraction line profile analysis. J. Appl. Crystallogr..

[36] Cheary R.W., Coelho A. (1992). A fundamental parameters approach to X-ray line-profile fitting. J. Appl. Crystallogr..

[37] Zhao Q., Zhang B., Hui W., Gao K., Ji H., Sun X., Feng X., Peng C., Wang K., Gao C. (2026). Buried 2D/3D heterojunction in n–i–p perovskite solar cells through solid-state ligand-exchange reaction. Nat. Energy.

[38] Ok A.C., Sarıoğlu C. (2024). Synthesizing of SnS_2_ photocatalyst from SnO_2_ powders by thermal sulfurization with varying temperature (400 °C and 500 °C) and time. Int. J. Hydrogen Energy.

[39] Pogue E.A., Goetter M., Rockett A. (2017). Reaction kinetics of Cu_2−x_S, ZnS, and SnS_2_ to form Cu_2_ZnSnS_4_ and Cu_2_SnS_3_ studied using differential scanning calorimetry. MRS Adv..

[40] Hobson T.D.C., Hutter O.S., Fleck N., Daniels L.M., Major J.D., Ng T.M., Durose K. (2020). Vegard Relation and Raman Band Reference Data Generated from Bulk Crystals of Kesterite-Phase Composition Series Cu_2_ZnSnS_4x_Se_4–4x_ (CZTSSe, 0 ≤ x ≤ 1). Cryst. Growth Des..

[41] Schorr S. (2011). The crystal structure of kesterite type compounds: A neutron and X-ray diffraction study. Sol. Energy Mater. Sol. Cells.

[42] Khan A.F., Mehmood M., Rana A.M., Bhatti M.T., Mahmood A. (2009). Optical Characterization of rf-Magnetron Sputtered Nanostructured SnO_2_ Thin Films. Chin. Phys. Lett..

[43] Fernandes P.A., Salomé P.M.P., da Cunha A.F. (2011). Study of polycrystalline Cu_2_ZnSnS_4_ films by Raman scattering. J. Alloys Compd..

[44] Baranov D., Lynch M.J., Curtis A.C., Carollo A.R., Douglass C.R., Mateo-Tejada A.M., Jonas D.M. (2019). Purification of Oleylamine for Materials Synthesis and Spectroscopic Diagnostics for trans Isomers. Chem. Mater..

[45] Isotta E., Mukherjee B., Fanciulli C., Pugno N.M., Scardi P. (2020). Order–Disorder Transition in Kesterite Cu_2_ZnSnS_4_: Thermopower Enhancement via Electronic Band Structure Modification. J. Phys. Chem. C.

