# Supplementary material for: Thermoelectric Generator Based on Kesterite (Cu2ZnSnS4) Synthesized via Sol–Gel Method
Source: Materials (Basel). 2026 May 10;19(10):1971. doi: 10.3390/ma19101971 (PMC13209125; doi:10.3390/ma19101971)
Supplement: Supplementary file 1 [file materials-19-01971-s001.zip › materials-4241046-supplementary.pdf]

Supplementary Information File: Thermoelectric Generator based on kesterite Cu_2ZnSnS_4 Synthesized via Sol-Gel Method

[1]Afef Tarhouni, [2]Marcelo Augusto Malagutti, [2]Tanguy Bernard, [2]Narges Ataollahi, [2]Eleonora Isotta, [3]Andrea Chiappini, [1]Hassen Dahman, [1]Lassaad El Mir, [2*]Paolo Scardi

[1] LaphyMNE Laboratory, Faculty of Sciences of Gabes, University of Gabes, Gabes 6072, Tunisia;

[2] Department of Civil, Environmental and Mechanical Engineering, University of Trento, Via Mesiano 77, 38123 Trento, Italy;

[3] CSMFO Laboratory, Fondazione Bruno Kessler (FBK) Photonics Unit, Institute of Photonics and Nanotechnologies (IFN-CNR), Via Alla Cascata 56/C, 39123 Trento, Italy;

Corresponding authors*: paolo.scardi@unitn.it

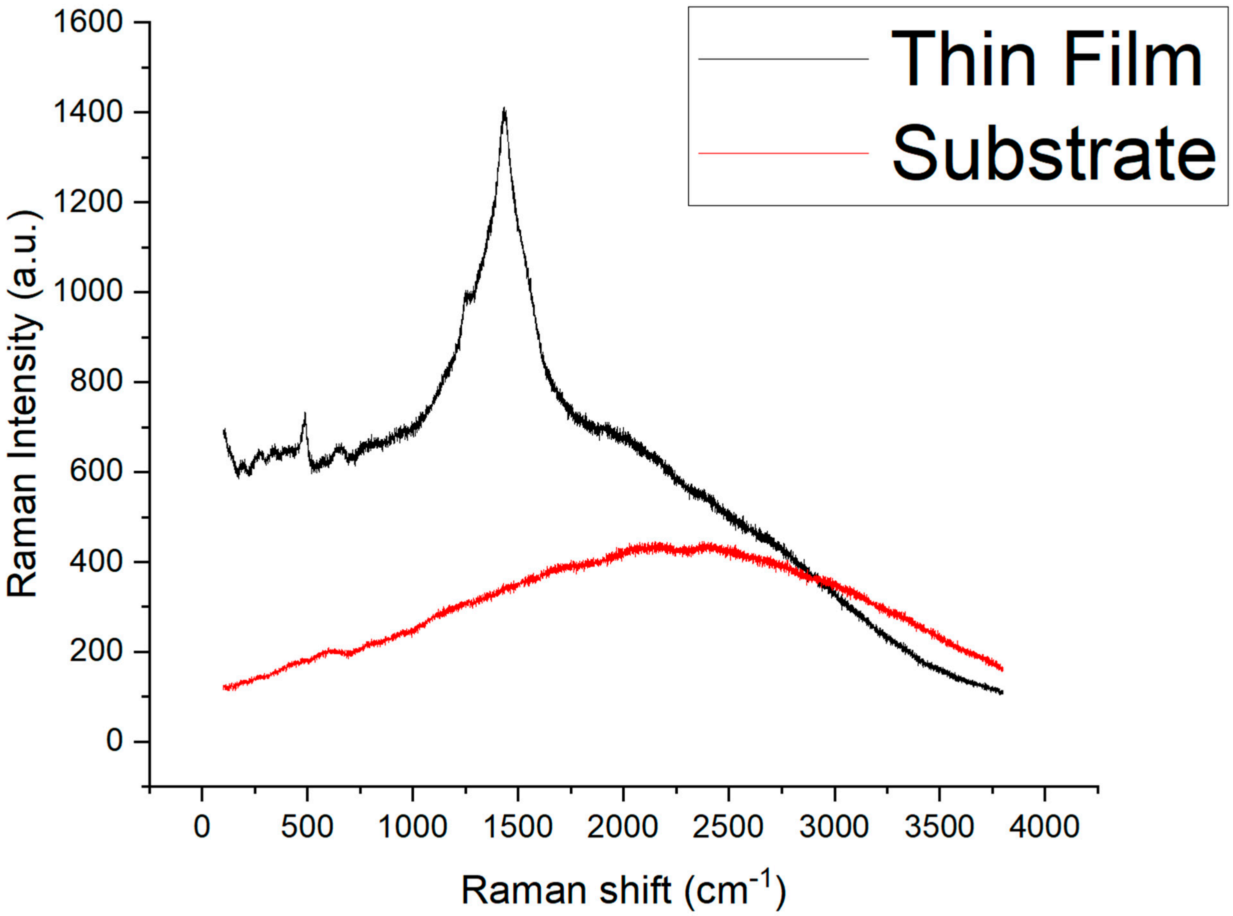

Figure S1. Raman spectra for the kesterite thin film (red line) and glass substrate (SiO_2, given by the black line).
